# Human behavior in free search online shopping scenarios can be predicted from EEG activation using Hjorth parameters

**DOI:** 10.3389/fnins.2023.1191213

**Published:** 2023-11-10

**Authors:** Ninja Katja Horr, Bijan Mousavi, Keren Han, Ao Li, Ruihong Tang

**Affiliations:** Brain Intelligence Neuro-Technology Ltd, Beijing, China

**Keywords:** decision-making, choice prediction, EEG, Hjorth parameters, machine learning

## Abstract

The present work investigates whether and how decisions in real-world online shopping scenarios can be predicted based on brain activation. Potential customers were asked to search through product pages on e-commerce platforms and decide, which products to buy, while their EEG signal was recorded. Machine learning algorithms were then trained to distinguish between EEG activation when viewing products that are later bought or put into the shopping card as opposed to products that are later discarded. We find that Hjorth parameters extracted from the raw EEG can be used to predict purchase choices to a high level of accuracy. Above-chance predictions based on Hjorth parameters are achieved via different standard machine learning methods with random forest models showing the best performance of above 80% prediction accuracy in both 2-class (bought or put into card vs. not bought) and 3-class (bought vs. put into card vs. not bought) classification. While conventional EEG signal analysis commonly employs frequency domain features such as alpha or theta power and phase, Hjorth parameters use time domain signals, which can be calculated rapidly with little computational cost. Given the presented evidence that Hjorth parameters are suitable for the prediction of complex behaviors, their potential and remaining challenges for implementation in real-time applications are discussed.

## Introduction

Human decision-making in real-life situations is complex and dependent on a myriad of known and unknown influencing factors. Based on intensive research, our understanding of logical reasoning, heuristics and biases that determine human decisions increased rapidly in the past decades (Johnson and Ratcliff, 2014; Benjamin, 2019). Still, combining all of these factors into realistic predictions of what will be the outcome of any individual choice, is a difficult endeavor. While choices in simple decision scenarios may be predicted via few environmental parameters (e.g., Brandstätter et al., 2006; Ge and Godager, 2021), such predictions become infinitely complex when looking into realistic choice settings from everyday life.

In order to approach the prediction of complex real-world decisions, it therefore makes sense to move from situational and cognitive factors to the brain, where all decision-making happens. Brain activation should, in theory, contain all the information necessary to predict any decision that will be made in the near enough future. However, the difficult question is, how the relevant activation can be identified and isolated from the large amount of noise, that is, activation which is uninformative regarding the decision at hand. The brain processes related to both simple and complex choices and how they interact to arrive at the final decision is an active field of research (Bault and Rusconi, 2020; Serra, 2021). Neuro-cognitive models have been developed to explain the brain processes behind decisions in laboratory experiments (e.g., Knutson and Bossaerts, 2007; Mulder et al., 2014; Tavares et al., 2017). However, such models may not be easily transferable to the complexity of real-world decision-making. Machine learning algorithms can, however, search the data for patterns and regularities which are related to a certain type of outcome, even without full understanding of the underlying processes. Therefore, applying machine learning to measurements of human brain activation may allow for the prediction of processes as complex as real-life human decision-making behavior.

The use of machine learning algorithms to increase the understanding of human brain functions and predict human behavior based on neural activation has become increasingly common in recent years (Vu et al., 2018). Low-level perceptual stimulus characteristics (e.g., Swisher et al., 2010; Schuck et al., 2015; Allen et al., 2021; Hajonides et al., 2021) and perceptual decisions (e.g., Sajda et al., 2009; Barik et al., 2019; Mercier and Cappe, 2020) can be predicted with often high levels of accuracy. There are also studies showing that more complex choice behavior, like choices based on learned environmental reward probabilities (e.g., Hampton et al., 2006), environmental exploration in order to seek information (e.g., Desender et al., 2019) and social decision-making (e.g., Speer and Boksem, 2019), can be decoded from brain activation.

In the present work, we focus on the prediction of purchase choices for basic consumer products. The prediction of purchase choices is an ongoing topic of marketing research and remains very challenging, despite a large amount of available information on both product and customers (Gal and Simonson, 2021). Similarly, neuromarketing research has not yet given sufficient evidence for specific neural correlates that robustly perform better than traditional methods in determining consumer preferences (Hakim and Levy, 2019). Regarding machine learning methods, some promising results for the prediction of purchase choices from brain activation have been reported. For example, Jai et al. (2021) report up to 95 percent prediction accuracy based on fMRI activation for purchase choices in an experimental setting. Mashrur et al. (2022) find up to 87 percent decoding accuracy with EEG. On the other hand, Garczarek-Bąk et al. (2021) concluded that EEG activation could not significantly improve the prediction of purchase choices compared to a prediction solely based on electrodermal activation (EDA) and product familiarity.

Besides aiming for a high prediction accuracy, we need to consider the requirements that make an algorithm predicting purchase choices via brain activation practically useful. Everyday life purchase choices are characterized by an extremely high variability in possible products and platforms. Therefore, general rules and heuristics cannot provide sufficient insights into how any given product should be presented. Rather, every single product or product line needs to be considered individually. The step-by-step manual adaptation of product presentation based on consumer research is a tedious process that only allows for a limited number and range of modifications to be made. Furthermore, while consumer profiles and explicit opinions based on buyer statistics or questionnaires can be obtained comparatively easily, cheaply and in large numbers, collecting brain activation data will always require more efforts. Therefore, to make the most out of brain activation data as an information source for consumer research, the development of algorithms that allow for real-time analysis is important. With real-time analysis future choice behaviors can be predicted during the decision process itself. On top of a faster turnaround on par with or even exceeding traditional consumer research measures, real-time prediction is the number one prerequisite for the qualitatively important step from a passive measurement of brain activation to an active feedback loop. Direct interaction between the potential customer’s brain activation and the product can be enabled through automated adjustment of the sensory input according to the outputs of a real-time algorithm. Individualized computer-generated product presentations can be created and the optimal presentation – the one which gives the highest probability of future purchase – can be determined for every given product and consumer.

With such future applications in mind, the present work attempts an EEG-based prediction of realistic purchase choices in an online shopping scenario. Participants are using their own devices and are spending their own money. Every product can be labeled as bought, put into the virtual shopping card or discarded (not bought). A range of commonly used machine learning algorithms is employed for predicting these labels. The method of EEG is an obvious choice when working with real-life decision-making, as it allows participants to interact naturally with their environment while providing a signal with high temporal resolution that can, via eye-tracking glasses and screen monitoring, be directly linked to participants’ momentary visual input (Horr et al., 2022).

A crucial question is, which aspect of the EEG signal should be used to train the algorithm. EEG measures electrical activation at the scalp with millisecond accuracy and a spatial resolution determined by the number of measurement electrodes or EEG channels (Jackson and Bolger, 2014). At each channel the signal can be decomposed into its frequency components, with a natural trade-off between time and frequency resolution (Cohen, 2011). While time-frequency decomposition is useful to identify neural correlates of behavior (Herrmann et al., 2016), it’s calculation is computationally intensive. This limits its applicability for rapid real-time predictions. A balance needs to be found between feature extraction that is sufficiently detailed to give accurate predictions and sufficiently simple for an algorithm whose speed and efficacy suits the application at hand. The time-frequency parameters described by Hjorth (1970) may be a feasible choice, both in terms of prediction accuracy and speed. Hjorth parameters describe the signal’s frequency characteristics in the time domain without the need to perform time-frequency decomposition. They are easy to calculate from the raw signal and can be stored as one signal vector per channel, rather than a time by frequency matrix. They therefore provide a much less complex basis for machine learning models of brain data than traditional measures, while still containing the basic temporal information of interest. These properties make Hjorth parameters promising features to focus on when building machine learning models based on brain data, especially when real-time prediction is of interest (e.g., Vidaurre et al., 2009; Mehmood et al., 2022; Rizal et al., 2022).

To our knowledge, the present study is the first to apply Hjorth parameters for the prediction of choices in a real-world scenario. Common linear and non-linear machine learning algorithms are trained and tested for their prediction accuracy to (1) determine the possibility to predict complex human decisions based on Hjorth parameters in a standard machine learning framework and (2) find out which algorithms and approaches are most suitable to predict such decisions. The present research should be considered as a first step toward the prediction of complex real-world decision-making in purchase situations. It can thereby serve as the basis for future improvements and for the development of brain-computer-interfaces (BCIs), that enhance product optimization by predicting online buying choices in real time.

## Methods

### Participants

Over a 3-year period, a total of 12 batches of participants were recorded in a realistic free browsing and free choice online shopping scenario. The main difference between the batches were the currently available and relevant product pages. The batches did not differ in study setup, location and equipment. Also, the general instructions remained the same for all batches. The batches did differ, however, in target products of interest and in the incentive given to participants. Table 1 summarizes participant characteristics for each of the batches. Data from a total of 391 individuals was collected, 262 (67%) of which were female and 129 (33%) were male. The average participant age was 29.0 years (*SD* = 5.3). All participants were naïve to the purpose of the study, had normal or corrected-to-normal vision and no history of neuropsychological diseases. Given every batch focused on a particular product type (e.g., certain types of hygiene products, cosmetics products, nutrition, and household products), pre-screening questionnaires ensured that the participants had a current intention to buy the respective product type. Participants received between 50 and 100 CNY/h for participation in the study as well as a up to 30% discount on all purchases. They gave their written consent after written and verbal explanation of task, procedure, measurement, and anonymized use of the data.

**Table 1:** Overview over participant characteristics in all batches.

Project date	*N*	Age				Gender	
		*M*	SD	Min	Max	Male	Female
01/05/2018	40	31	2.7	26	36	14	26
01/06/2018	4	24	1.6	21	25	0	4
01/08/2019	30	26	4.8	19	35	7	23
01/10/2019	78	30	5.4	19	45	31	47
11/11/2019	11	27	4.7	19	35	4	7
12/12/2019	51	27	4.4	18	35	16	35
01/01/2020	47	29	6.0	19	40	15	32
01/04/2020	31	29	7.4	19	43	13	18
01/06/2020	30	30	4.8	21	39	10	20
01/08/2020	26	29	5.0	18	38	7	19
11/11/2020	15	28	3.4	22	34	3	12
12/12/2020	28	28	4.3	20	35	9	19

Participant characteristics are shown separated by batch with each batch focusing on different target products and therefore different available product pages. The general procedure was the same for each batch.

### Data collection

All data were collected at the EEG laboratory of Brain Intelligence Neuro-Technology Ltd. In an electrically shielded room, participants’ EEG, eye movements and field of vision were recorded while they used their personal phone to browse online shopping websites. A study session lasted up to 1.5 h, including about half an hour for equipment set up and subsequent free browsing without a fixed time limit. Eye movement and field of vision recordings were done using SMI eye tracking glasses (SensoMotoric Instruments GmbH, Teltow, Germany). EEG was recorded with an EasyCap system (EasyCap GmbH, Herrsching-Breitbrunn, Germany).

The EEG cap consisted of 63 Ag/AgCl electrodes placed at the standard locations of the international 10/10 system. Position AFz served as the ground electrode and position FCz as the reference electrode. One additional external electrode was placed under the right eye. Electrode impedance was kept below 5 kΩ throughout the course of the experiment. The sampling rate for signal digitization was 500 Hz. After the recording the signal was re-referenced to the average over all non-rejected measurement electrodes.

Upon completion of the setup 1 min of resting state activation was recorded during which participants sat still with their eyes open. Then participants were instructed to take out their phone and use an online shopping application of their choice to search for a particular type of product. They were told to spend as long as they like browsing through the application and buying their products of choice.

**Figure 1:**
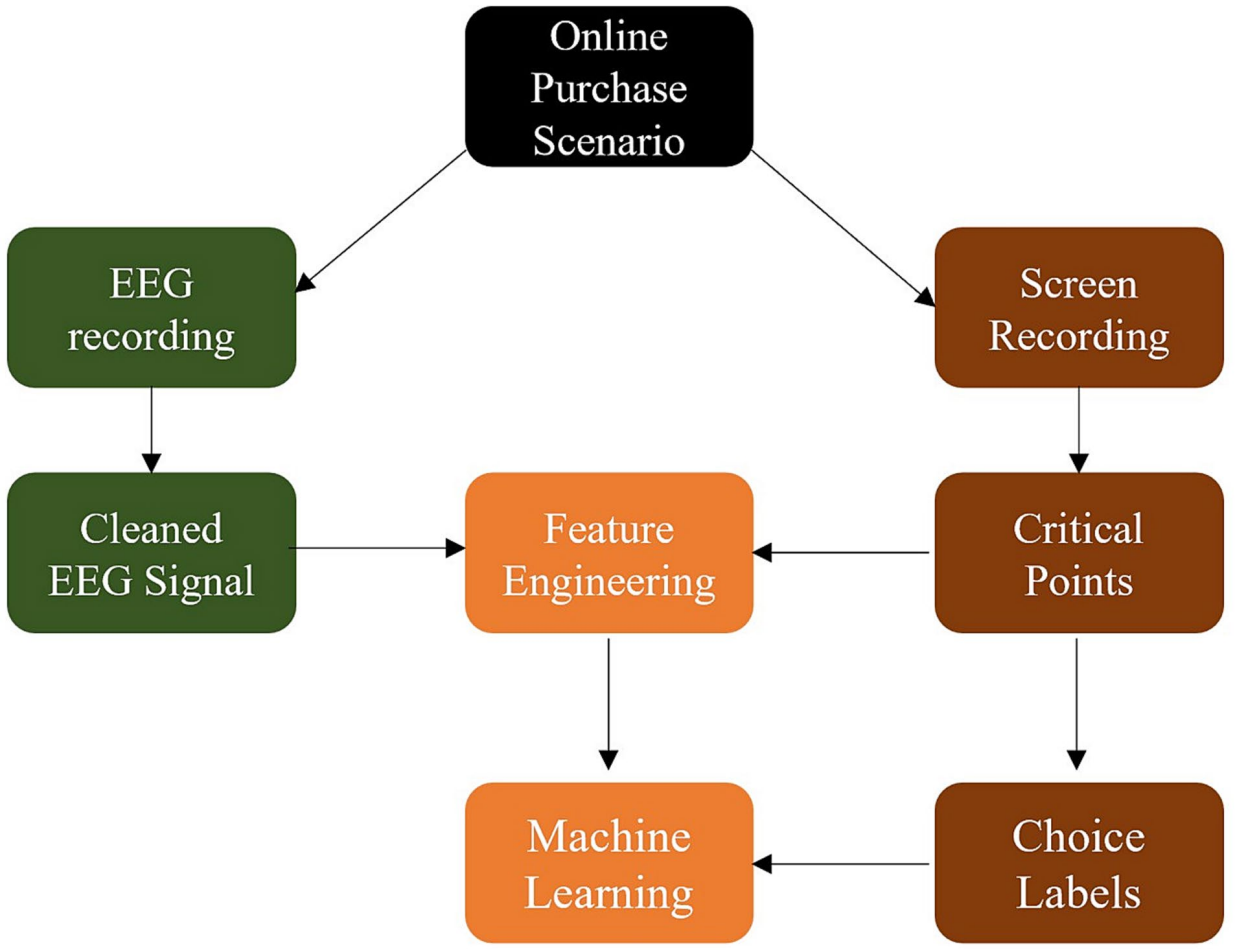
Overview of collected data and modeling rationale. The available data can be divided into EEG recordings and field-of-vision screen recordings relating EEG activation to the real-world online shopping scenario. The screen recordings were used to choose critical points in time and label the EEG time snippets according to their buying status. Features extracted from EEG activation around the critical points in time were used for prediction of the buying status.

All products were bought from participants’ own money and shipped to their own addresses making the study situation a realistic online shopping scenario. As noted above, varying types of products were the focus of the different batches and varying amounts of buying incentives were guaranteed. An overview over the collected data and their use for building prediction models can be seen in Figure 1.

### Data analysis

#### EEG pre-processing

Matlab R2017B (The MathWorks, Natick, Massachusetts) and the Matlab-based software package Fieldtrip (Oostenveld et al., 2011) were used for pre-processing. A 0.5 Hz highpass and 48–52 Hz bandpass filter were applied over the full signal.

Principal component analysis with a logistic infomax ICA algorithm was employed to remove eye-artifacts (Makeig et al., 2002). Further EEG artifacts were rejected via visual inspection. Episodes containing artifacts over all channels were removed. Rejected channels were interpolated using the average of their neighboring channels weighted by distance.

#### Data segmentation and tagging

**Figure 2:**
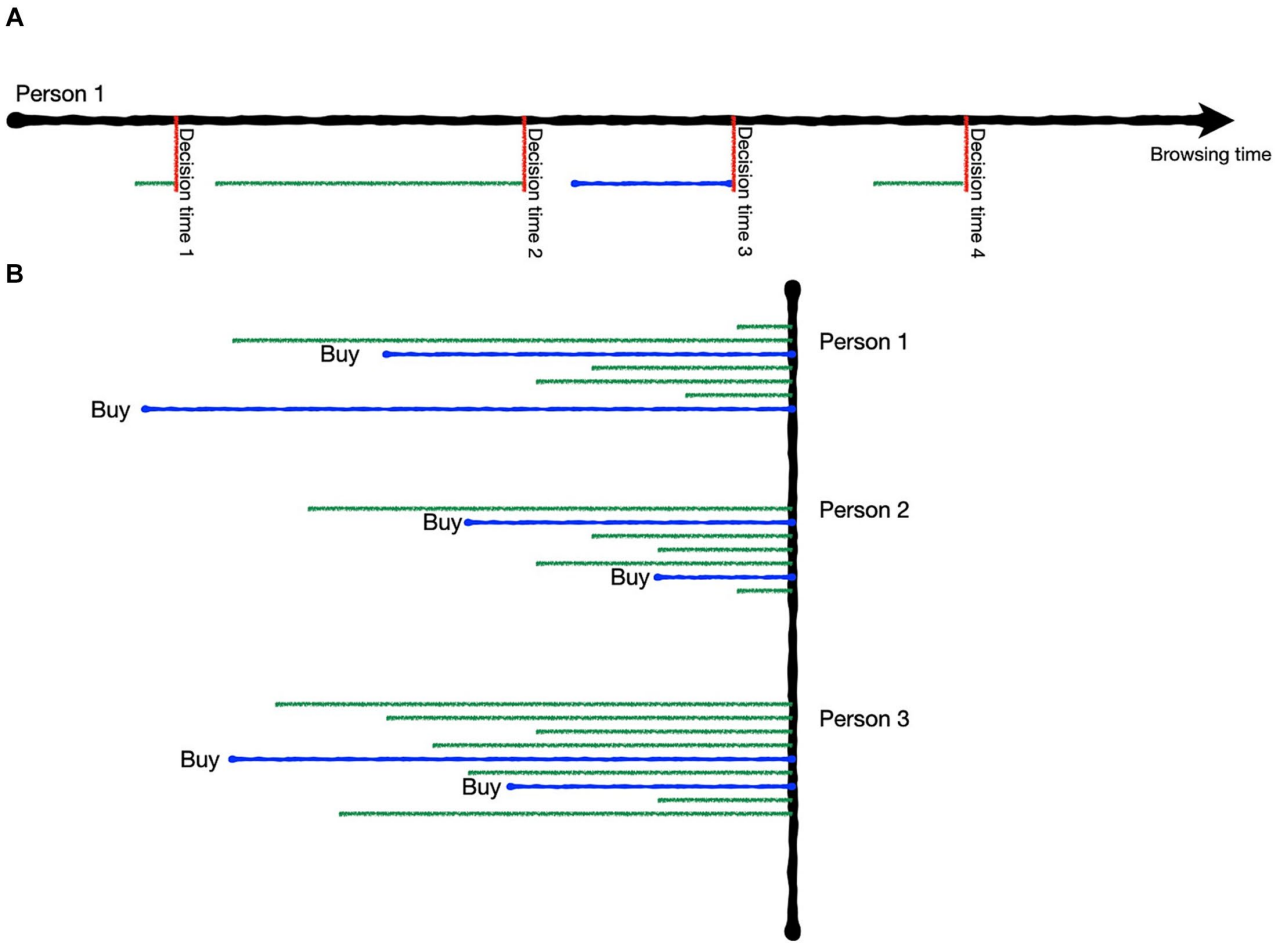
Illustration of the data segmentation. **(A)** Each subject’s browsing time was manually marked at each point a product page was entered or left. The time the product page was left is deemed the decision time (vertical red lines), i.e., the point at which the current decision about the product is definitely made. **(B)** Each decision time was labeled according to the buying status of the product and the full duration of the product viewing was available for each label (horizontal green and blue lines = examples of product page views for each participant; green = no-buy, blue = buy).

The EEG signal was cut into episodes with each episode marking the complete time span during which an individual product page was viewed without interruption. Entering and leaving of each product page was marked manually based on the field of vision recordings. As the leaving of the page was considered the point when the final decision for this view of the given product has been reached, it was termed decision time and used as the reference point for the time snippets analyzed. The starting point was only used to determine the maximum time available for each product page. A visual illustration of the segmentation process is shown in Figure 2. Each episode of viewing a product page was tagged according to the buying status regarding that product. The tags defined the labels the prediction algorithm had to forecast. We used two different approaches of data tagging – a two-class and a three-class approach.

The two-class approach simply differentiated between the labels buy and no-buy. All episodes of viewing products that have either been bought or put into the shopping cart at any point in time during the study session were labeled as buy episodes. All others were labeled as no-buy episodes. The rationale behind combining buy and cart episodes was that both are assumed to represent a positive attitude toward the product and the general willingness to buy it (Horr et al., 2022).

The three-class approach differentiated between buy, cart and no-buy episodes. All episodes of products that are never purchased or put into the cart were tagged as no-buy. As soon as a product was put into the cart all later and previous episodes of that product were tagged as cart. As soon as a product was bought all later episodes and all previous episodes up until the point when the product has been put into the cart were marked as buy. For instance, if a product was viewed six times, added to the cart during the third view and purchased during the fifth view, the tagging would have been [c, c, c, b, b, b] with c representing cart and b representing buy. The three-class approach is less focused on the general opinion or approach tendency toward the product and more on the practical outcome of the decision. The backward labeling with episodes from the same product being labeled first as card and then as buy assumed that the time before a decision marks activation in favor of this decision, while as long as no further decision is recorded the general stance toward the product stays the same. As opposed to the two-class approach, the three-class approach made it possible that the very same product page belonged to a different category within one person with the only differentiation being the participants’ subsequent decision regarding the product.

#### Data augmentation

To increase the number of data samples that can be used for training the prediction model and to take human error into account, four samples of equal duration were created for each time span representing a tagged product page view. To model error performance, we let the operators mark start and end points of at least 500 product pages twice. The maximum error turned out to be in the range of 200 msec. Taking this calculated maximum human error into account, each decision point was taken to start at t – 200 msec, t – 100 msec, t and t + 100 msec, respectively. This led to four differently timed samples of 100 msec duration: [t – 200, t – 100], [t – 100, t], [t, t + 100], and [t + 100, t + 200].

Though adding more augmented data, that is, splitting the signal up into more time spans, would further increase accuracy, this comes at the expense of calculation complexity and time. Splitting up each signal into four therefore was found to be a good balance. Note that as opposed to common practices, for example in image processing, the augmentation here is based on real data simply differing in the selected window of time.

**Figure 3:**
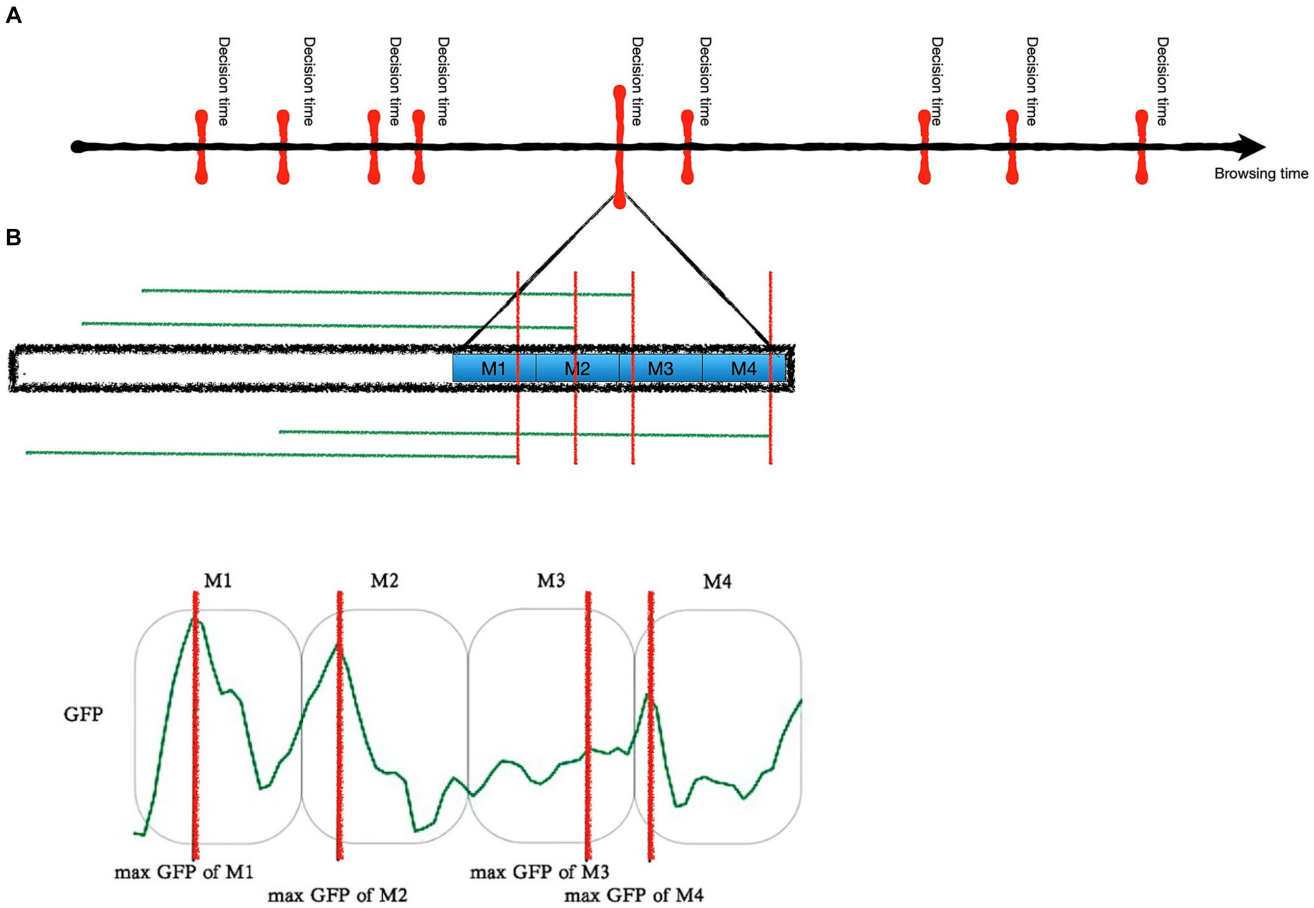
Illustration of the data augmentation and extraction of maximum GFP. **(A)** For each decision time four samples were created. Each sample contained the activation of all channels, but fixing the decision time at a slightly different time point to (1) augment the data with additional samples and (2) take human error of a maximum of 200 msec into account. **(B)** From each sample Hjorth parameters at the time of maximum global field power (GFP) were used for prediction.

For each thus generated time window, maximum global field power (GFP) was chosen as the critical time point within the given time span and was used for further feature engineering. It has been shown that points of maximum GFP are the ones with the highest signal-to-noise ratio (Damborská et al., 2019). The choice of maximum GFP as the critical time point was therefore based on practical considerations regarding extractable information from the signal rather than theoretical considerations of which kinds of information represent decision-making. The data augmentation and extraction of GFP is illustrated in Figure 3.

#### Feature engineering

At each critical point of highest global field power Hjorth parameters were extracted from the time-domain EEG signal for time periods denoted by τ over all 61 channels. While the full duration of each product page viewing was theoretically available, it was attempted to use the minimal duration of data sufficient to predict decisions. Minimal data has a lower processing complexity, which means that the speed of the algorithms is faster and less expensive hardware is required for sufficiently rapid real-time predictions. Prediction was attempted with time snippets τ of 1,200, 1,400, 1,600, 1,800, and 2,000 msec around the point of highest global field power. 1 × 61 brain feature maps of the Hjorth mobility and the Hjorth complexity parameter for each time period τ were used to classify purchasing behavior.

Since Bo Hjorth's, 1970 conception (Hjorth, 1970), Hjorth parameters have been used for time-domain signal processing as identifiers of statistical properties in a multitude of applications (e.g., Mouzé-Amady and Horwat, 1996; Leite and Moreno, 2018). Hjorth parameters have also frequently been used to extract information about the temporal characteristics of EEG signals (e.g., Elbert et al., 1992; Cecchin et al., 2010; Mehmood and Lee, 2015). In EEG applications they are referred to as normalized slope descriptors (NSDs, Mouzé-Amady and Horwat, 1996).

The advantage of Hjorth parameters is that they can directly extract frequency moments from the time domain of the signal without having to undertake transformations into the frequency domain. This is of particular interest when building machine learning algorithms and brain-computer interfaces, as it has been shown to allow the prediction of complex states with relatively simple modeling approaches (e.g., Vidaurre et al., 2009; Mehmood et al., 2022; Rizal et al., 2022). Due to the computational efficiency of both the data transformation and the applied models, Hjorth parameters are promising features for developing algorithms that can make rapid real-time predictions from brain activation recorded via EEG.

The three Hjorth parameters are activity, mobility, and complexity (Hjorth, 1970). The Hjorth activity parameter (1) describes the zero ***-*** order moment of the frequency domain representing the variance of the frequency amplitude. The Hjorth mobility parameter (4) is the second order moment (2) divided by the first order moment (1) of the frequency domain and denotes the power spectrum’s mean frequency. The Hjorth complexity parameter (5) is the fourth order moment (3) divided by the second order moment (2) of the frequency domain and quantifies the variation in frequency. This parameter compares the signal to a pure sine wave with a value of 1 indicating a perfect match.


(1) $$m_{0}=\mathrm{HjorthActivity}=\frac{1}{T}\int_{t-T}^{t}f^{2}\left(t\right)dt$$

(2) $$m_{2}=\frac{1}{T}\int_{t-T}^{t}{\left(\frac{df}{dt}\right)}^{2}dt.$$

(3) $$m_{4}=\frac{1}{T}\int_{t-T}^{t}{\left(\frac{d^{2}f}{dt^{2}}\right)}^{2}dt$$

(4) $$\mathrm{Hjorth}\;\mathrm{Mobility}=\sqrt{\frac{m_{2}}{m_{0}}}$$

(5) $$\mathrm{Hjorth}\;\mathrm{Complexity}=\sqrt{\frac{m_{4}}{m_{2}}}$$

The input signal for the calculation of Hjorth parameters as features for the present models was the artifact-corrected raw EEG signal with a timespan of τ around the point of highest GFP. Due to the augmentation four raw EEG signal snippets were available for each product page. Hjorth parameters were calculated separately for each channel resulting in a 61-channel feature vector of parameter values for each snippet. In order to ensure a consistent value range across all snippets and increase model robustness against outlier values, a min-max normalization scaling the values to a range between 0 and 1 was applied over feature vectors (Singh and Singh, 2020). Such normalized Hjorth mobility and Hjorth complexity feature vectors were used to train the algorithm.

### Prediction model

Python 3.8 and the Python-based toolbox scikit-learn 1.0.2 (Pedregosa et al., 2011) were used for modeling. We employed and compared a range of simple machine learning algorithms to predict buying status on the basis of Hjorth mobility and Hjorth complexity brain feature maps. Initially, 80 percent of the data was used for training the model and 20 percent was used as the test set. Prediction accuracies were calculated based on 5-fold cross-validation. In a second step, snippets with the lowest possible tau were reanalyzed using a leave-one-subject-out cross-validation procedure, for which accuracies were calculated as the average accuracy over all subjects. The latter validation ensured, as opposed to the former, that there could never be augmented variants of the same product page view both in the training and in the test set. In the following each of the employed machine learning algorithms compared is shortly described. Wherever not otherwise specified the default settings of the scikit-learn version 1.0.2 were used.

#### Linear discriminant analysis

Linear discriminant analysis (LDA) seeks to classify the outcomes of a categorical target variable by dividing it in N-dimensional feature space through linear combinations of the dependent variables. The predicted category for each sample (buy, no buy or cart) is determined based on its position in feature space.

#### Logistic regression

Logistic regression (LR) is another method that allows for the prediction of categorical target variables as a linear combination of independent variables. Here, the continuous log odds of the target variables are predicted from the linear combinations of predictors. The log odds are transferred back into the probability of one compared to another category (e.g., 70% probability of buying compared to 30% of no-buying) and the more likely category will be predicted by the model.

#### K-nearest neighbors (k-NN)

The k-nearest neighbors (k-NN) technique divides the data into categories based on the similarity between samples. Different *k* values, that is, number of neighbors accounted for, were tried for the present algorithms. As *k* = 5 outperformed the others, the result of *k* = 5 is the one presented here.

#### Support vector machines

Support vector machines (SVM) are used to classify data by locating a hyperplane in N-dimensional feature space. Along with linear classification, SVMs can perform efficient non-linear classification using a technique called the kernel trick (Ben-Hur et al., 2008), which involves implicitly mapping their inputs into high-dimensional feature spaces. SVMs with linear, polynomial, and radial basis function kernels (RBF) were applied on the present data. The SVMs with RBF kernels performed the best for classification based on complexity and are therefore the ones reported. For mobility the SVM algorithms did not reach convergence.

#### Random forest

Random forests (RF), alternatively called random decision forests, are an ensemble learning technique for classification that utilizes a large number of decision trees to train. A random forest model determines the predicted category by the one that the majority of trees in the classification task selected. Random forests allow to identify which feature contributes the most to the classification accuracy, that is, for the present data it could be used to determine which EEG channels contributed most to making accurate predictions.

## Results

### Descriptive results

**Figure 4:**
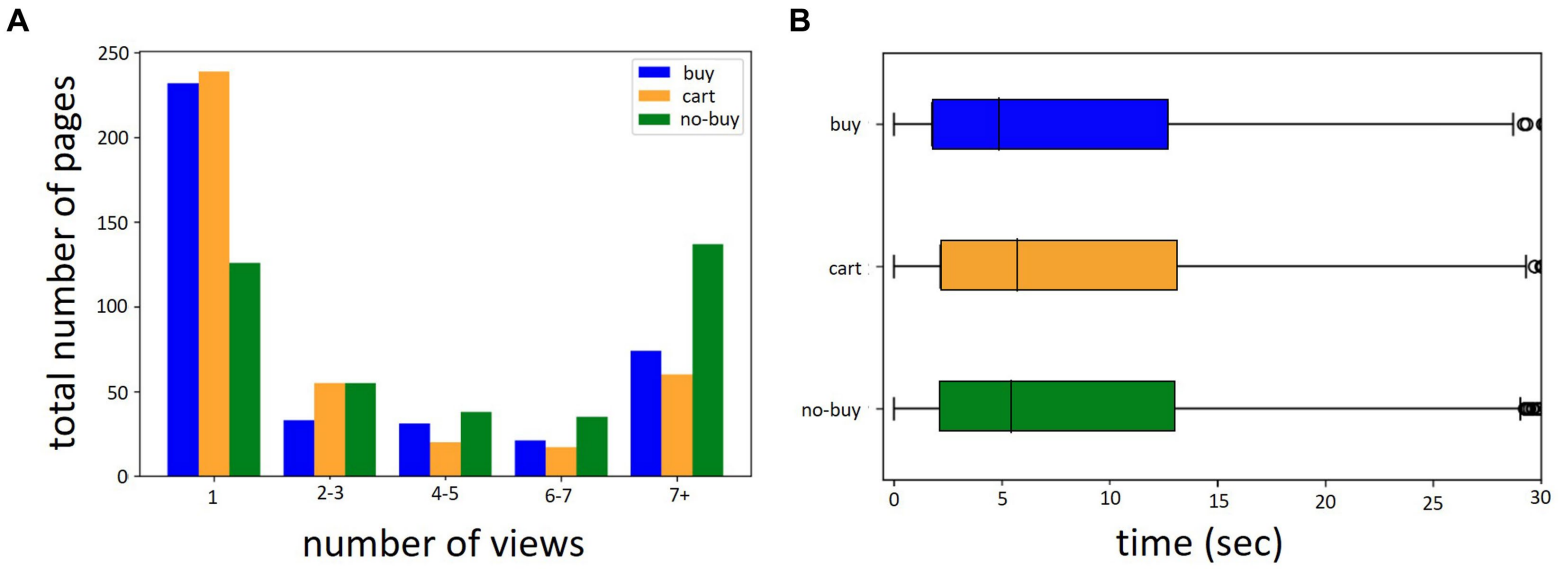
Number of pages viewed and viewing times. **(A)** Distribution of the number of pages by number of views (Total *N* pages = 6,030). **(B)** Distribution of viewing time in seconds. Both **(A,B)** are separated by labeling category.

**Figure 5:**
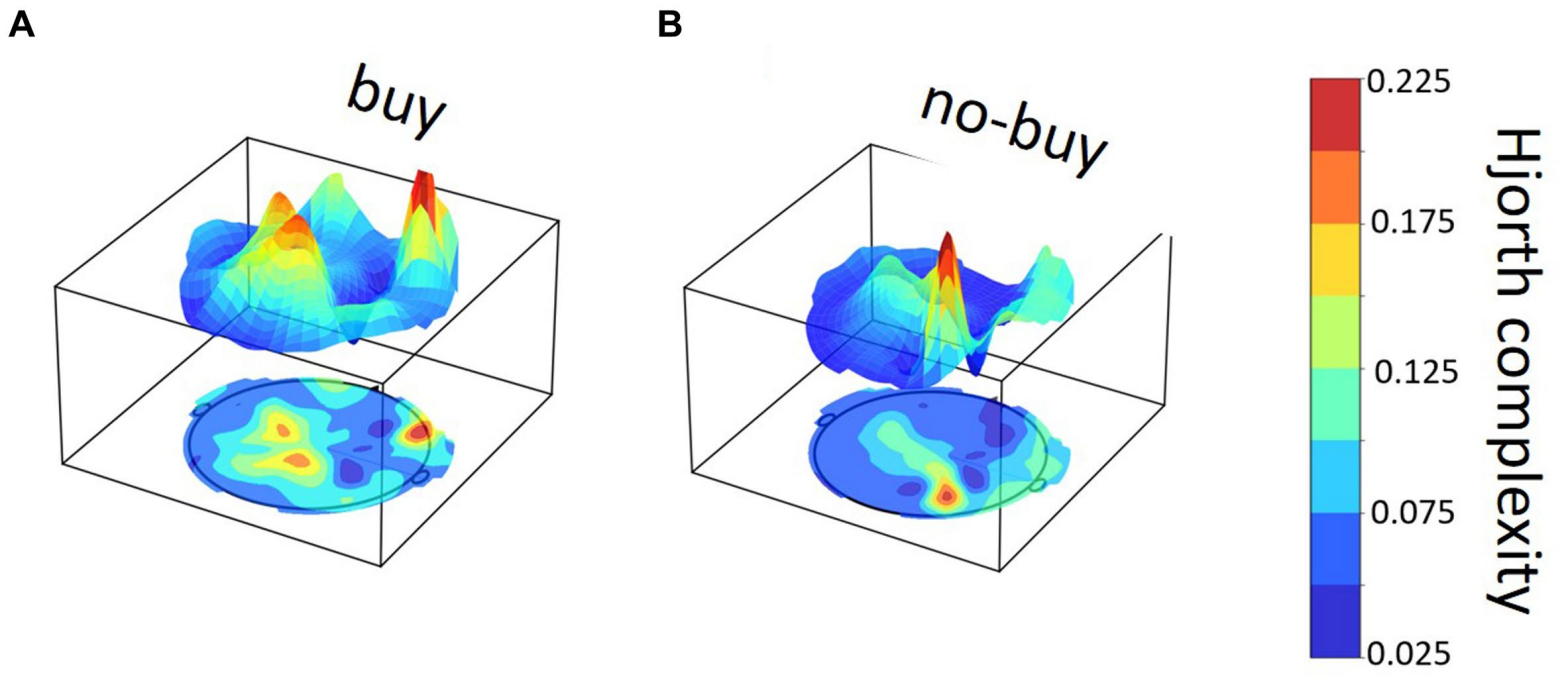
Exemplary Hjorth complexity brain map for one buy and one no-buy product page view. Example of Hjorth complexity distribution across the brain in a single product page view for **(A)** a buy and **(B)** a no-buy view in the same participant.

**Figure 6:**
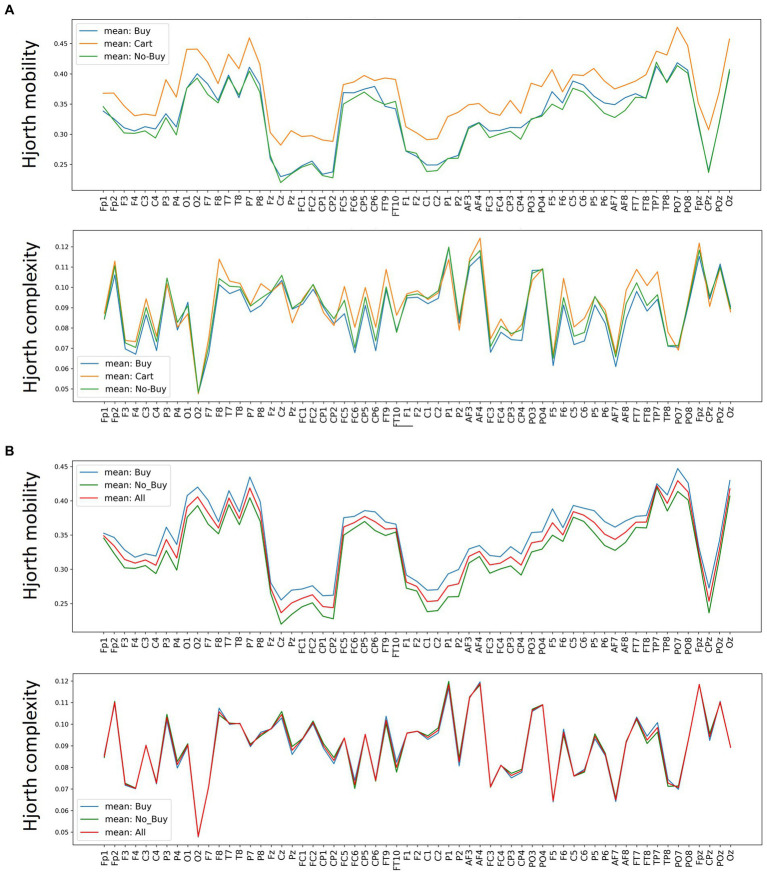
Average Hjorth mobility and complexity per channel. Average Hjorth mobility and Hjorth complexity for each EEG channel over all trials and subjects, seperated by **(A)** buy, cart and no-buy for the 3-classification case and **(B)** buy, no-buy and merged over all trials for the 2-classification case.

Over all 12 batches and 391 participants a total of 6,030 product pages were viewed (*M pages per participant* = 8.38, *SD* = 11.52, *Min* = 1, *Max* = 123). 24% were bought, 25% were put into the shopping cart and 51% were discarded. Per participant the average number of bought pages was *M* = 4.05 (*SD* = 6.54, *Min* = 0 *Max* = 47). Of cart pages it was *M* = 4.11 (*SD* = 9.22, *Min* = 0, *Max* = 123) and of not bought pages *M* = 8.84 (*SD* = 11.35, *Min* = 0, *Max* = 85). Mean viewing time for an individual page was *M* = 9.82 s (*SD* = 10.89, *Min* = 0.23, *Max* = 40). The mean viewing time for products over multiple pages was *M* = 31.83 s (*SD* = 12.70, *Min* = 1.06, *Max* = 40). An overview over the viewed number of pages by the number of times the page has been viewed and the labeling categories buy, cart and no-buy can be seen in Figure 4A. Figure 4B shows the distribution of viewing times for the three labeling categories. Participants spend an average of *M* = 13.46 min (*SD* = 12.57, *Min* = 0.64, *Max* = 81.56) browsing for products. Hjorth mobility and complexity of product page views were calculated separately for each EEG channel at the point of maximum GFP around the decision time of interest. An example of a Hjorth Complexity brain map for a single buy and single no-buy product page view can be seen in Figure 5. Figure 6A shows the average Hjorth mobility and Hjorth complexity values for the 3-class classification and Figure 6B for the 2-class classification.

### Prediction of buying behavior

#### Hjorth mobility

Table 2 summarizes the balanced classification accuracies using Hjorth mobility to differentiate between three classes (buy, cart and no buy). The performance of the linear discriminant analysis using 5-fold cross-validation (CV) is identical to random selection for all values of τ between 1,200 and 2000 msec (*M* = 33.0%, *SD* = 0%). With Leave-one-subject-out CV the accuracy almost doubles to 62%. The mean balanced accuracy of the logistic regression algorithm is only slightly above chance with 5-fold CV (*M* = 45.8%, *SD* = 3.91%) and even worse with leave-one-subject-out CV (34%). These poor results of linear classification approaches indicate that the feature distribution in N-dimensional space with *N* = 61 channels is non-linear, so that a planar hyperplane is not sufficient to accurately classify the data. The non-linear SVM performs equally poorly over all τ-coefficients both with 5-fold CV (*M* = 48.0%, *SD* = 0.0%) and leave-one-subject-out CV with τ = 1,200 msec (38%). Note that these results can be attributed to the SVM algorithm not reaching convergence. The k-NN algorithm shows an only slightly better average prediction accuracy over all τ-coefficients in 5-fold CV (*M* = 65.0%, *SD* = 0.63%) as well as leave-one-subject-out CV (61%). The random forest model results in by far the best prediction accuracies for all algorithm variants (5-fold cross-validation: *M* = 89.60%, *SD* = 0.49%; leave-one-subject-out CV = 82%). It can be seen that there is no systematic improvement of prediction accuracy due to adding more history, that is, longer τ-coefficients, to the data. The leave-one-subject-out CV shows worse prediction accuracies for all but the LDA algorithm – however, predictions can be considered more valid, as augmented samples based on the same product page view (as well as the same subject) can never be both in the training and in the test set.

**Table 2:** Prediction accuracy for 3-class classification with the mobility feature.

Model	20–80% 5-fold CV τ in msec					Leave-one-subject-out CV τ in msec
	1,200	1,400	1,600	1800	2000	1,200
	Prediction accuracy in %					Prediction accuracy in %
LDA	33	33	33	33	33	63
LR	47	47	48	48	48	34
k-NN	64	64	65	65	65	61
SVM	48	48	47	48	48	38
RF	88	88	89	89	89	82

LDA, linear discriminant analysis; LR, logistic regression; k-NN, k nearest neighbor; SVM, support vector machine; RF, Random Forest; CV, cross-validation. Algorithm parameters as described in the methods section.

The prediction accuracies for the Hjorth mobility two-class classification results are presented in Table 3. Similar as in the case of 3-class classification linear algorithms showed an insufficient prediction accuracy close to chance with 5-fold CV *M* = 53.2% (*SD* = 0.74%; leave-one-subject-out CV = 61%) for LDA and *M* = 58.5% (*SD* = 0.40%; leave-one-subject-out CV = 57%) for logistic regression. SVM performed equally poorly and without convergence (5-fold CV M = 57.6%, SD = 0.55%, leave-one-subject-out CV = 57%). The non-linear k-NN approach reached a decoding accuracy of *M* = 74.6% (*SD* = 0.80%: leave-one-subject-out CV = 67%). The by far best classification accuracy was again found for the random forest model with *M* = 91.8% (*SD* = 0.40%; leave-one-subject-out CV = 86%).

**Table 3:** Prediction accuracy for 2-class classification with the mobility feature.

Model	20–80% 5-fold CV τ in msec					Leave-one-subject-out CV τ in msec
	1,200	1,400	1,600	1800	2000	1,200
	prediction accuracy in %					prediction accuracy in %
LDA	54	54	53	53	52	61
LR	58	59	59	59	58	57
k-NN	75	74	76	74	74	67
SVM	57	58	58	57	58	57
RF	91	92	92	92	92	86

LDA, linear discriminant analysis; LR, logistic regression; k-NN, k nearest neighbor; SVM, support vector machine; RF, random forest; CV, cross-validation. Algorithm parameters as described in the methods section.

#### Hjorth complexity

For most algorithms, Hjorth complexity shows similar results to Hjorth mobility for both the 3-class (see Table 4) and the 2-class (see Table 5) classification. In the 3-class classification, linear algorithms still show the worst prediction accuracy with *M* = 38.0% (*SD* = 0%; leave-one-subject-out CV = 64%) for LDA and *M* = 48.0% (*SD* = 0.63; leave-one-subject-out CV = 37%) for logistic regression. Also, the k-NN approach shows similar prediction accuracies for Hjorth complexity as for Hjorth mobility (*M* = 62.4%, *SD* = 0.49%; leave-one-subject out CV = 67%). A clear improvement is seen regarding the prediction accuracy of the support vector machine algorithm (*M* = 88.2%, *SD* = 0.40%; leave-one-subject-out CV = 86%), which, now converging, is superior compared to the other three algorithms and is almost on par with the barely changed prediction accuracy of the random forest model (*M* = 89.0%, *SD* = 0%; leave-one-subject-out CV = 86%). Higher τ values always lead to similar or higher prediction accuracies. The improvement due to τ, however, is very small. In the successful algorithms (SVM and RF) the less error-prone leave-one-subject-out procedure leads to a slight decrease in prediction accuracies.

**Table 4:** Prediction accuracy for 3-class classification with the complexity feature.

Model	20–80% 5-fold CV τ in msec					Leave-one-subject-out CV τ in msec
	1,200	1,400	1,600	1800	2000	1,200
	Prediction accuracy in %					Prediction accuracy in %
LDA	38	38	38	38	38	64
LR	47	48	48	48	49	37
k-NN	62	62	62	63	63	61
SVM	88	88	88	88	89	79
RF	89	89	89	89	89	82

LDA, linear discriminant analysis; LR, logistic regression; k-NN, k nearest neighbor; SVM, support vector machine; RF, random forest; CV, cross-validation. Algorithm parameters as described in the methods section.

**Table 5:** Prediction accuracy for 2-class classification with complexity feature.

Model	20–80% 5-fold CV τ in msec					Leave-one-subject-out CV τ in msec
	1,200	1,400	1,600	1800	2000	1,200
	prediction accuracy in %					prediction accuracy in %
LDA	58	58	57	57	58	59
LR	58	58	58	58	58	58
k-NN	73	73	72	73	74	67
SVM	90	91	91	91	90	86
RF	91	91	92	92	92	86

LDA, linear discriminant analysis; LR, logistic regression; k-NN, k nearest neighbor; SVM, support vector machine; RF, random forest; CV, cross-validation. Algorithm parameters as described in the methods section.

For two-class classification LDA (*M* = 57.6%, *SD* = 0.48%; leave-one-subject-out CV = 59%) and logistic regression (*M* = 58%, *SD* = 0%; leave-one-subject-out CV = 58%) again show the worst performance. Also, the *k* nearest neighbor performance does not differ much between complexity and mobility (*M* = 73.0%, *SD* = 0.63%; leave-one-subject-out CV = 67%). Compared to Hjorth mobility, a strongly improved performance is again found for the support vector machine (*M* = 90.6%, *SD* = 0.49%; leave-one-subject-out CV = 86%), which now converges. The random forest model (*M* = 91.6%, *SD* = 0.49%; leave-one-subject-out CV = 86%) shows no improvement for complexity compared to mobility and is now on par with the support vector machine algorithm.

#### Channel importance

**Figure 7:**
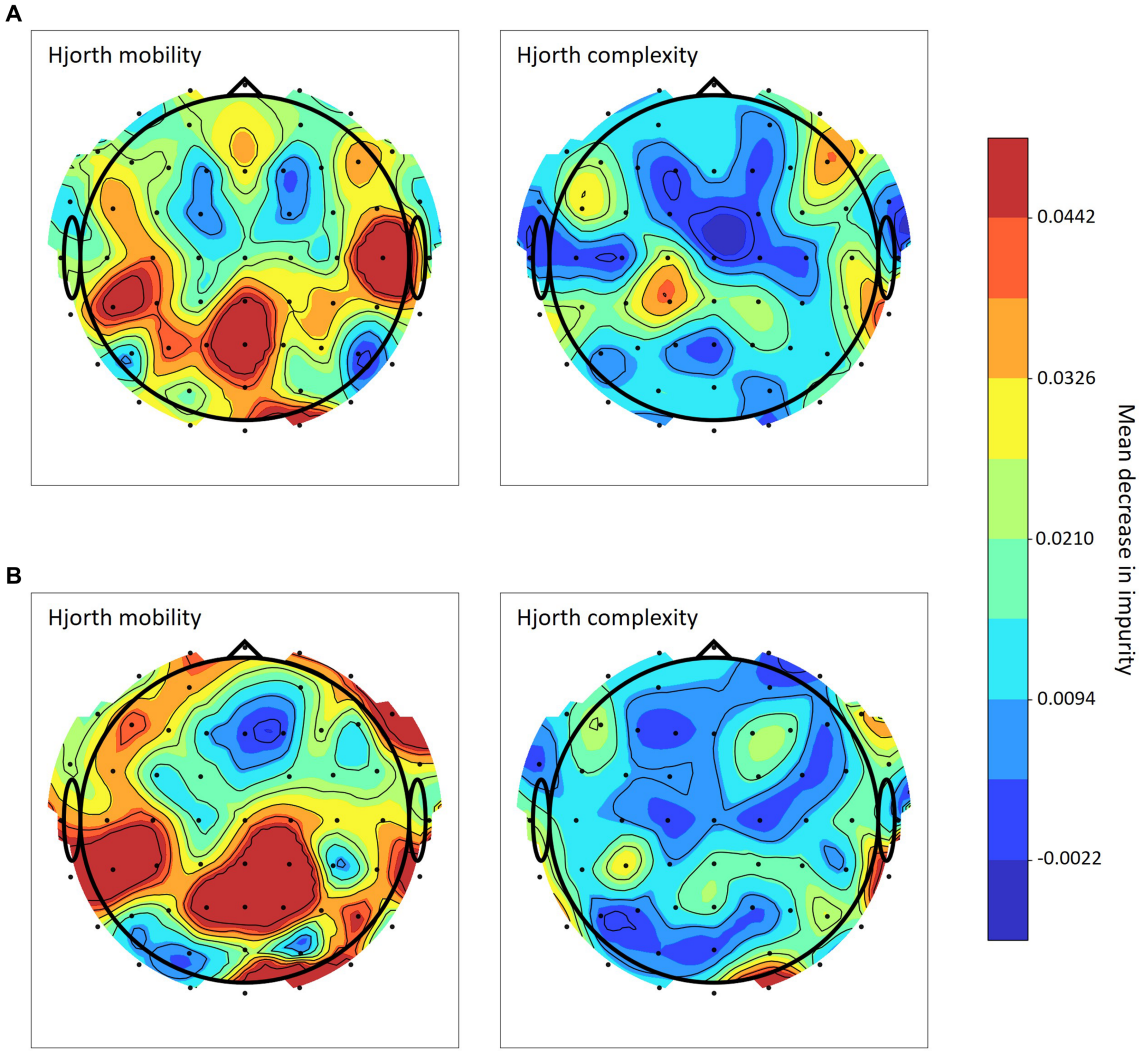
Random forest channel importance based on Hjorth mobility and complexity Note. Channel importance calculated via mean impurity decrease in the random forest model for **(A)** 3-class classification and **(B)** 2-class classification with τ = 1,200 msec.

The random forest model led to the best decoding accuracies in both decoding approaches (2-class and 3-class), for both mobility and complexity as well as across all τ-coefficients. Using a random forest model, it can be calculated which channels contributed most to category predictions. Figure 7A illustrates the channels with the greatest effects for Hjorth mobility and for Hjorth complexity in the 3-class classification approach and Figure 7B in the 2-class classification approach. Feature importance is depicted for both 3-class and 2-class classification mobility and complexity with τ equal to 1,200 msec. The mean and standard deviation of the impurity decrease within each tree are used to determine the feature importance (Altmann et al., 2010). The advantage of such permutation-based feature importance is that it is not biased toward features with a high cardinality, i.e., many unique values, and is suitable to be computed on a left-out test set.

#### Comparison to time-frequency-based prediction

To allow for a direct comparison between Hjorth-parameter and time-frequency based prediction, the 2-class and 3-class classification with the reported machine-learning algorithms was repeated using alpha-power (8–13 Hz) and theta-power (5–7 Hz) gained from traditional time-frequency decomposition based on Morlet-wavelet convolution with 7 wavelet cycles. This led to prediction accuracies below 65% in both the 2-class and the 3-class classification for all algorithms. The above frequency bands were chosen, as they were previously shown to be related to purchase choices in the present online shopping scenario (Horr et al., 2022). Of course, adding additional time-frequency features may still result in a better prediction. However, the decomposition of the EEG signal into its complete frequency representation is much more computationally demanding than the calculation of Hjorth parameters (Hjorth, 1970; Vidaurre et al., 2009; Rizal et al., 2022).

## Discussion

The present study used EEG activation from realistic online shopping scenarios to predict participants’ purchase choices. Predictions were based on Hjorth mobility and Hjorth complexity (Hjorth, 1970) during the viewing of product pages that were categorized either into bought, put into cart and not bought products or only into bought and not bought products. A range of common machine learning algorithms were employed to determine these choice categories. Applying leave-one-subject-out cross-validation choices could be predicted with an accuracy up to 82% in the 3-class classification and 86% in the 2-class classification.

Differences between different τ durations as investigated with a 20%/80% 5-fold cross-validation were minimal for all algorithms, features, and classification approaches (see Tables 2–5). This suggests that the lowest investigated τ-coefficient in the present study, 1,200 msec, is already sufficient to get the most out of the present prediction approach. Leave-one-subject-out cross-validation lead to slightly lower prediction accuracies than 5-fold cross-validation. However, due to augmentation, only the leave-one subject out, but not the 5-fold CV ensures that product page views in the training set are not repeated in the test set. Therefore, the former is considered more robust and its results are in the following reported as the final accuracies reached.

Comparing the performance of different prediction algorithms with τ = 1,200 msec and a leave-one-subject out cross-validation (see Tables 2–5), it is obvious, that linear algorithms were not sufficient to distinguish between the different choice categories. The accuracy of the linear algorithms, i.e., linear discriminant analysis and logistic regression stayed below 65% for all classification conditions. The lack of a clear linear differentiability between categories is also apparent in the average mobility and complexity values shown in Figure 6. The non-linear k-nearest neighbor approach also reached poor mean prediction accuracies around 60%. For Hjorth mobility, a random forest model clearly showed the best results. With 82% decoding accuracy in the 3-class and 86% in the 2-class classification respectively, it was consistently at least 10 percentage points better than the second-best algorithm, k-NN. Interestingly, while the support vector machine algorithm did not converge for Hjorth mobility, its prediction accuracies caught up with the random forest models when using Hjorth complexity. With Hjorth complexity both the support vector machine and the random forest model reached an accuracy of around 80% in the three-class and above 85% in the two-class classification.

The present results demonstrate that random forest models based on Hjorth mobility or complexity as well as support vector machines with RBF kernels based on Hjorth complexity can decode buying decisions during the viewing of a product page with a remarkable accuracy above 80%. As the present analysis was done after the recording, computation time was only of minor importance. However, in order to be able to transfer this kind of decoding into a real-time application scenario, that is, to make a prediction about the decision during the viewing of the page and allow for direct interaction between brain activation and product presentation, time will become a major concern. Regarding features, the time required to calculate Hjorth complexity is much greater than the time required to calculate Hjorth mobility. This speaks for random forest models, which also performed well with Hjorth mobility, to be the most promising method in future applications. Certainly, it will be beneficial to have a large number of possible classifiers available for future applications to select the most suitable one based on situation-specific constraints regarding accuracy-time trade-offs.

It should be noted that all present results are based on augmented samples, that is, making four samples out of each product page by means of a 200 msec temporal jitter. Furthermore, the choice of the critical time point as the point of highest GFP was crucial for achieving the presented decoding accuracies. Without augmentation and GFP for time selection, prediction performance did not exceed 65% in any of the algorithms. Under the given conditions, mobility and complexity have been identified as suitable features for the prediction of real-world decisions. In comparison, alpha and theta frequency power did not allow for prediction accuracies above 65%. While features based on complete frequency decomposition may have led to a better performance of time-frequency data, their calculation requires far more resources in terms of time and computational power (Hjorth, 1970; Vidaurre et al., 2009; Rizal et al., 2022). It remains to be determined by future research whether additional features based on dynamical systems theory may be similarly or even more promising both in terms of performance and speed.

As a limitation of the present approach, it must be pointed out, that machine learning models based on summarized brain features like Hjorth parameters make it difficult to determine, which aspect of the brain signal and even more so which cognitive component is the basis of the algorithm’s prediction accuracy. This can also be seen in the strong variability of feature importance for different random forest models. Such variability suggests that situation- and person-specific prediction models are necessary for a reliable prediction as no general pattern can be extracted. It was ensured that the prediction algorithm is based on the brain signal itself rather than artifacts (e.g., movement artifacts) related to the buying decision by comparing categories for which no different observable behavior is to be expected. That is, for the entire product page viewing time span no difference in general behavior and movement between buying and no-buying can be assumed. Furthermore, Hjorth parameters were calculated around the point of highest global field power (GFP), which is most robust against artifacts (Damborská et al., 2019). However, besides the algorithm being based on temporal components of the EEG signal, it can give little insights into the neural correlates of the cognitive processes leading to the decision. The prediction of purchase choices should therefore be seen as a practical approach to shine light on decision outcomes, enabling the understanding of product preferences and respective optimization.

While the present results were calculated after collection and storage of the data, the most interesting practical applications to decision decoding in realistic scenarios require real-time prediction. Only real-time prediction allows to move away from long iterations of data collection, analysis, redesign and new data collection toward automated presentation adjustment and thereby product- and consumer-specific optimization in a single session. In the present example this means that the purchase choice should be predicted during the viewing of the product page in advance of any decision being made. Such an actual prediction of the choice to be made enables an algorithm-driven optimization of the presentation toward the highest probability of buying. Real-time prediction is therefore the first step to develop a brain-computer-interface (BCI) of product optimization. Such a device would, on top of a much quicker turnaround, have two important qualitative advantages compared to decoding buying choices after the recording session and making manual adjustments. (1) A brain-computer-interface would remove limitations on the number and range of possible product presentations to be tested. This is because, finding the optimal presentation is no longer being based on human trial and error, but constitutes a computational optimization problem. While manual adjustment requires the pre-selection of specific presentation designs, a BCI algorithm could choose from a practically unlimited parameter space of possible designs, with the goal of finding the best, that is, most likely to be bought, product presentation. (2) With manual adjustment it is practically impossible to test both a large number of potential consumers and a large number of different presentation designs within the same consumer. As it does not make sense to go through the tedious process of manual adjustment for individual consumers, manual optimization of the product presentation must be based on average consumer preferences, ignoring or at least overshadowing differences between consumers. In a BCI-scenario, however, optimization takes place on the level of the individual consumer – so that each session produces the best possible design for the study participant at hand. In combination with easily attainable information on the participant, like demographics and general buying habits, this allows for detailed customer segmentation, targeting different product presentations at identified clusters of potential customers.

In order to make real-time prediction possible, several adjustments to the present analysis strategy are necessary. Besides optimizing the algorithms to require as little computing power and time as possible, also the entire pre-processing pipeline needs to be integrated into real-time computation. Here, the start and end points of product page views were determined by human operators. Human error calculated to be about 200 msec was considered within the segmentation process. However, an automatic evaluation of the eye-tracking video based on computer vision analysis (see, e.g., Voulodimos et al., 2018; Cazzato et al., 2020; Chai et al., 2021) could reduce, if not completely eliminate, this error and, of course, is the only possible solution for real-time prediction of decisions. Algorithms for automatic EEG data cleaning are available and show consistent improvement (Rashmi and Shantala, 2022). While the present data cleaning was again dependent on human operators, future work remains to establish to what extent the same decoding accuracies can be achieved based on automatically cleaned signals. In order to allow real-time prediction, it is further important to use pre-processing approaches that do not, like for example independent component analysis, require the full signal. Another option may be to develop algorithms which are suitable to work with the EEG raw signal, so data cleaning is not necessary for high accuracy prediction.

Furthermore, for fast and efficient real-time prediction, it would be advantageous to reduce the number of channels used by the algorithm. In random forest models channel importance (see Figure 7) can be employed to determine which channels are the most suitable because they most strongly influence the algorithm’s final prediction. However, in the present analysis channel importance varied a lot depending on the Hjorth parameter, prediction approach and even the τ-coefficient being used. This suggests that the optimal algorithm for a particular use case first needs to be determined on the basis of all channels and then tested for the most promising subsets.

Another practical question for both post-hoc and real-time prediction concerns the labels to be classified. We here distinguished between a two-class and a three-class classification approach showing that both reached reasonable and in the best models even very similar absolute decoding accuracies. Still, there will always be a general trade-off between the detail of classification and final classification accuracy. In order to determine the necessary level of detail in each use case, it should therefore be considered, what the different labels mean in terms of the underlying cognitive processing and expected action steps to follow. Regarding the example of the two-class versus three-class approach, one could speculate, that the two-class classification better distinguishes between general interest and no interest in the product, while three-class classification is more suitable if the immediate action following the viewing of the product is of interest. An even more specific labeling based on the exact stage of a potential buyer’s decision process is possible and may lead to higher ecological validity of the outcome categories. For example, in the present study, all views of a product that took place before and after it was put into cart were labeled as “cart” in the 3-class classification and all views that took place before and after a product was bought were labeled as “buy” in both the 2-class and the 3-class classification. However, actions that might indicate reconsideration, like removing an item from the cart or revisiting a page after the product has been bought, were not labeled separately. Therefore, a possible change of opinion was not represented in the present class labels. While adding such a change of opinion as a classification category would probably represent the actual cognitive processing of participants better, the increase in categories and decrease in number of cases for each category will necessarily decrease decoding accuracies.

For further improvement of the presented algorithms both in terms of speed and accuracy additional features in the field of dynamical systems theory can be explored (Rodríguez-Bermúdez et al., 2015; Gao et al., 2020; Parbat and Chakraborty, 2021). Furthermore, person-related features like demographics (e.g., age, gender, education), personality traits and general as well as session-specific data on shopping behavior might lead to additional improvements. Different ways of determining the optimal decision time for prediction may also increase both speed and accuracy as well as shed light on the particularly interesting question, how far in advance of the action the decision is predictable with a certain level of accuracy.

In conclusion, the present study demonstrates the possibility of predicting complex decisions in a real-world scenario based on EEG parameters. Interestingly, simple and commonly used non-linear machine learning algorithms were sufficient to make reasonable predictions above 80% decoding accuracy. It therefore seems feasible to move from the presented prediction approach taking place after data collection and storage to real-time prediction of complex decision-making and automated adjustment of stimulus presentation. In order to do so, future research needs to improve the given algorithms in terms of speed and independence from human operators, while increasing and stabilizing decoding accuracies. For practical applications in different use cases, it must further be investigated which EEG features and algorithms are most suitable for different kinds of real-world prediction scenarios.

## Data availability statement

The raw data supporting the conclusions of this article will be made available by the authors, without undue reservation.

## Ethics statement

Ethical approval was not required for the studies involving humans because the studies were not conducted in association with a university, i.e., there was no dedicated ethics committee. The studies were conducted in accordance with the local legislation and institutional requirements. The participants provided their written informed consent to participate in this study.

## Author contributions

NH, KH, and RT designed the study. BM and KH analyzed the data. NH wrote the manuscript. AL Writing – review and editing, Methodology, Formal analysis, and Visualization. All authors reviewed and revised the submitted manuscript.
